# Dynamic Fracture and Crack Arrest Toughness Evaluation of High-Performance Steel Used in Highway Bridges

**DOI:** 10.3390/ma16093402

**Published:** 2023-04-26

**Authors:** William N. Collins, Tristan D. Yount, Ryan J. Sherman, Roberto T. Leon, Robert J. Connor

**Affiliations:** 1 Department of Civil, Environmental and Architectural Engineering, University of Kansas, Lawrence, KS 66045, USA; tyount19@ku.edu; 2 School of Civil and Environmental Engineering, Georgia Institute of Technology, Atlanta, GA 30332, USA; ryan.sherman@ce.gatech.edu; 3 The Charles Edward Via, Jr. Department of Civil and Environmental Engineering, Virginia Polytechnic Institute and State University, Blacksburg, VA 24061, USA; rleon@vt.edu; 4 Lyles School of Civil Engineering, Purdue University, West Lafayette, IN 47907, USA; rconnor@purdue.edu

**Keywords:** impact energy, fracture toughness, master curve, stress intensity rate, high-performance steel

## Abstract

Impact energy tests are an efficient method of verifying adequate toughness of steel prior to it being put into service. Based on a multitude of historical correlations between impact energy and fracture toughness, minimum impact energy requirements that correspond to desired levels of fracture toughness are prescribed by steel bridge design specifications. Research characterizing the fracture behavior of grade 485 and 690 (70 and 100) high-performance steel utilized impact, fracture toughness, and crack arrest testing to verify adequate performance for bridge applications. Fracture toughness results from both quasi-static and dynamic stress intensity rate tests were analyzed using the most recently adopted master curve methodology. Both impact and fracture toughness tests indicated performance significantly greater than the minimum required by material specifications. Even at the AASHTO Zone III service temperature, which is significantly colder than prescribed test temperatures, minimum average impact energy requirements were greatly exceeded. All master curve reference temperatures, both for quasi-static and dynamic loading rates, were found to be colder than the Zone III minimum service temperature. Three correlations between impact energy and fracture toughness were evaluated and found to estimate reference temperatures that are conservative by 12 to 50 °C (22 to 90 °F) on average for the grades and specimen types tested. The evaluation of two reference temperature shifts intended to account for the loading rate was also performed and the results are discussed.

## 1. Introduction

Testing Charpy V-Notch (CVN) impact specimens has been standard practice for material qualification in the structural steel industry for over a century. The sampling, fabricating, and testing of CVN specimens are both cost- and time-efficient. While impact energy absorption is not a direct measure of fracture toughness, a multitude of correlations between impact energy and fracture toughness have been developed [1,2,3,4,5,6]. One, the Barsom and Rolfe Two-Stage Correlation [1], was used to form the basis for the minimum impact energy and test temperatures specified in the AASHTO LRFD Bridge Design Specification (BDS) [7]. Structural steels that meet these minimum impact energy requirements are believed to have an acceptable level of fracture toughness without the need for more complex fracture toughness tests.

To measure fracture toughness, two main specimen geometries are commonly used: compact [C(T)] and single-edge bend [SE(B)]. These specimens are much more cost- and time-intensive than CVNs but utilize elastic–plastic fracture mechanics concepts in calculating a single value parameter that describes the fracture behavior of a material in the presence of a crack. Generally represented as a critical fracture toughness parameter, *K_c_* represents the ability of a material to resist crack initiation. Similarly, the arrest toughness, *K_a_*, measures a material’s ability to arrest a crack that has already begun to propagate. As with impact testing, fracture toughness and crack arrest testing are often performed at a range of temperatures representing minimum service temperatures for the desired material application, which is often well below room temperature. For the ferritic steels being examined herein, each of the tests mentioned results in lower shelf behavior in terms of absorbed impact energy or toughness at cold temperatures, characterized by brittle cleavage fracture. At elevated temperatures, ductile tearing is the dominant failure mechanism, resulting in an upper shelf of material behavior. The area in between these two shelves is known as the transition region. Marked by increased scatter and mixed failure modes, failure in this region is often the result of small amounts of ductile tearing that reach a carbide or other inclusion that acts as an initiation site for cleavage fracture.

A popular method of analyzing fracture toughness data is the master curve method. The master curve, developed by Wallin [8,9] and codified in ASTM E1921 [10], is an exponential curve that describes the fracture behavior of ferritic materials in the lower transition region. A statistics-based approach that uses a three-parameter Weibull distribution to present the cumulative probability of failure, the shape of the curve is unchanged regardless of toughness. Instead, the location of the median probability curve with respect to temperature is described by the reference temperature, *T_o_*. This is defined as the temperature at which the median curve is equal to 100 MPa√m (91 ksi√in).

In addition to being performed at a range of temperatures, fracture toughness testing can be performed at a range of stress intensity rates. Fracture toughness test standards ASTM E1820 [11] and E1921 [10] provide guidance for testing at quasi-static, intermediate, and dynamic stress intensity rates. As the testing rate increases, fracture toughness has been shown to decrease [9]. The inverse is also true, and the change in fracture toughness caused by rate effects can be represented by shifting the toughness transition curve along the temperature axis. A reference temperature adjustment, such as the one developed by Wallin [9] and verified by others [12,13], can be used to compare fracture toughness data obtained at various testing rates. Correlations between impact energy and fracture toughness must also take the rate effect into consideration, as impact testing is dynamic by nature. In the case of the Barsom and Rolfe Two-Stage Correlation, the rate effect is considered using a yield-stress-dependent temperature shift.

Material fracture characterization often utilizes both impact energy and fracture toughness testing. Studies focused on characterizing the fracture behavior of high-performance steel (HPS) evaluated CVN impact energy and quasi-static fracture toughness utilizing SE(B) specimens [14,15,16,17]. The primary goal of these studies was to characterize high-toughness materials currently used in highway bridge applications to potentially reduce the frequency and rigor of in-service inspection requirements. However, as highway traffic applies loads at rates significantly higher than a quasi-static rate, understanding material behavior at elevated loading rates is important. Additionally, the potential for a material to arrest a fracture prior to system failure is a topic of recent interest in the highway bridge community. Presented herein are the results and detailed analyses of both dynamic rate and crack arrest toughness data. The master curve method, as defined by the currently adopted version of the fracture toughness test standard ASTM E1921 [10], is used to analyze the fracture and arrest toughness results. The stress intensity rate reference temperature adjustment mentioned above is used to compare quasi-static and dynamic fracture toughness data. Multiple correlations between impact energy and master curve reference temperature are also evaluated. Results indicate that all quasi-static and dynamic reference temperatures were well below minimum service temperatures for highway bridge structures. In contrast, crack arrest reference temperatures did not reach service temperatures and were found to be closer to current CVN impact test temperature requirements. Finally, a temperature adjustment equation has been proposed, allowing for the estimation of crack arrest reference temperature based on quasi-static fracture initiation data.

## 2. Materials and Methods

HPS steel was developed in a joint effort between the Federal Highway Administration (FHWA), the American Iron and Steel Institute (AISI), and the United States Navy with the specific goal of developing a high-toughness, weldable steel for bridge applications [18]. Early investigations of HPS indicated the material exhibited enhanced fracture behavior and fabrication capabilities compared with conventional bridge steels [19]. Further studies focused on CVN impact energy and quasi-static fracture toughness testing, as well as the testing of full-scale structural components [20,21,22,23]. However, no studies of HPS have evaluated fracture behavior at elevated loading rates. To fill that gap in knowledge, CVN and Charpy-sized SE(B) specimens were sampled from eight plates of various thicknesses of ASTM A709 [24] grades HPS 485 W and 690 W (70 W and 100 W). Crack arrest specimens were also sampled from three of the eight plates. The plates were given letter designations for specimen sampling and testing purposes. Plates A, D, H, I, and J were HPS 485 W (70 W), while Plates C, E, and F were HPS 690 W (100 W). Plate information, including letter, grade, thickness, and number of each specimen type are contained in Table 1 and are consistent with the presentation of results in Collins et al. [17]. It should be noted that the number of specimens tested is shown outside of the parentheses, while only valid data, as reported by Collins [14], are counted within the parentheses. Additionally, only CVN specimens sampled from the plate quarter-thickness in the L–T orientation are included in this analysis and counted below, as these are directly comparable to the fracture toughness test results.

**Figure 1:**
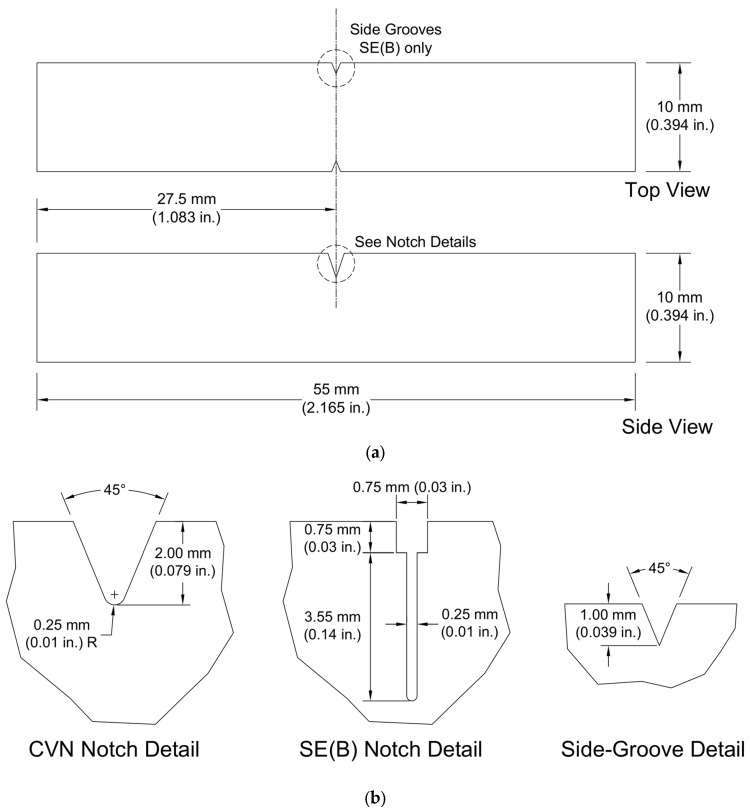
(**a**) Top and side views and (**b**) notch and side-groove details of CVN and SE(B) specimens.

In accordance with ASTM A673 [25], both CVN and Charpy-sized SE(B) specimens were sampled from the quarter-thickness location, with the exception of Plate C specimens, which were sampled from the third-thickness due to insufficient plate thickness. The specimens were oriented such that the fracture propagated perpendicular to the rolling direction of the plate, commonly referred to as the L–T orientation. Both specimen types were fabricated as full-size Charpy blanks with geometry shown below in Figure 1a.

CVN notches were cut with a manual broach to a nominal depth of 2 mm (0.079 in), leaving an 8 mm (0.315 in) ligament as the critical dimension. Impact specimens use a blunted notch, while fracture toughness testing requires a sharp crack front. Prior to cleavage testing, a sharp crack is achieved using cyclical fatigue pre-cracking. To ease the fatigue crack initiation, a narrow notch was cut with an electrical discharge machine (EDM) to the width of the wire used, in this case 0.25 mm (0.01 in). Along with the 4.3 mm (0.169 in) deep notch, shoulders were machined into the specimen to facilitate the attachment of a clip gage to measure crack mouth opening displacement (CMOD) during pre-cracking and testing. Because fatigue crack growth is typically reduced at free surfaces due to a lack of constraint, 1 mm (0.039 in) deep side grooves were machined after pre-cracking and prior to cleavage fracture testing to provide a straight crack front and eliminate any effects of crack tunneling. Notch details for both specimen types and side-groove details are shown in Figure 1b.

### 2.1. Charpy V-Notch Impact Testing

Impact tests were performed using a friction-compensated Tinius Olsen impact hammer with a capacity of 400 J (300 ft-lbf). Specimens were tested at a range of temperatures to create a full temperature transition curve. For test temperatures between 21 and −80 °C (70 and −112 °F), specimens were submerged in a bath of methanol prior to testing. In the event that a well-defined lower shelf was not apparent at −80 °C (−112 °F), liquid nitrogen was used to achieve lower temperatures. Specimens were placed in an insulated container along with a companion specimen that was instrumented with an embedded thermocouple to monitor the temperature. The temperature of the test specimen was allowed to decrease below the desired test temperature, at which point the test specimen and companion specimen were removed from the liquid nitrogen and placed on a metal plate. Once the thermocouple measurement reached the test temperature, the impact test was performed.

The temperature–toughness transition curve is commonly represented by a five-parameter sigmoid curve fit to the data [26]. The expression for the curve, *CVN(T)*, used herein is provided in Equation (1):

(1) $$CVN\left(T\right)=a+\frac{b}{{\left(1+e^{\frac{-\left(T-c\right)}{d}}\right)}^{m}}$$

where *a* is the asymptotic minimum, *b* is the asymptotic maximum, *c* is the location parameter, *d* is the slope parameter, *m* is the scale parameter, and *T* is the test temperature.

### 2.2. Fracture Toughness Testing

Fracture toughness testing was carried out in accordance with ASTM E1921 [27] using Charpy-sized SE(B) specimens tested on an MTS Landmark servo-hydraulic load frame. Pre-cracking was performed in two steps using a three-point bend apparatus in accordance with ASTM E647 [28]. The span of the setup was four times the specimen width, or 40 mm (1.575 in). The first pre-crack step used a stress intensity of 17.5 MPa√m (16.0 ksi√in), while the second step used a stress intensity of 14.3 MPa√m (13.0 ksi√in). Specimens were rotated between pre-cracking steps to avoid uneven crack growth due to misalignment. Fatigue cracks were grown until the initial crack length, *a_o_*, reached a nominal length of one-half of the specimen width, 5 mm (0.197 in), resulting in a fatigue crack length of 0.7 mm (0.028 in). At this point, side grooves were machined on the faces on either side of the notch, centered on the specimen in line with the fatigue crack.

The same three-point bend apparatus was used for fracture toughness testing. Similar to the CVN testing, the fracture toughness tests were performed at an array of sub-ambient temperatures. Liquid nitrogen was used to cool an environmental chamber that surrounded the bend fixture and specimen. Specimens were instrumented with a thermocouple connected to a process controller, which controlled the flow of liquid nitrogen through a cryogenic solenoid valve. In this manner, the specimen temperature was able to be regulated to within ±1 °C (±1.8 °F) of the test temperature. Once the specimen reached the desired temperature and prior to testing, multiple elastic loading and unloadings were performed to ensure that the specimen was seated properly and that the clip gage CMOD reading was accurate. For cleavage testing, load was applied in displacement control. The quasi-static tests used a nominal stress intensity rate of 0.1 MPa√m/s (0.09 ksi√in/s), while dynamic stress intensity rates ranged from 1000 to 5000 MPa√m/s (910 to 4550 ksi√in/s). At the completion of each test, the specimens were heat tinted to ease differentiation between fatigue and cleavage surfaces. Load-CMOD records and optically measured crack sizes were then used to calculate the critical J-integral at the moment of failure, *J_c_*, which includes both plastic and elastic components.

The total area under the load-CMOD curve, *A*, is comprised of an elastic component, *A_e_*, and a plastic component, *A_p_*. The area is divided into the two components based on the reciprocal of the slope of the initial elastic loading, called the compliance, *C_o_*. The plastic component of the J-integral, *J_p_*, is calculated by evaluating the plastic area, *A_p_*. Elastic fracture toughness, *K_e_*, is calculated based on specimen geometry and load at fracture, and it is subsequently converted into the elastic component of the J-integral, *J_e_*. Summing *J_e_* and *J_p_*, the resulting *J_c_* can then be converted into an elastic–plastic equivalent critical fracture toughness, *K_Jc_*, using Equation (2):

(2) $$K_{Jc}=\sqrt{J_{c}\frac{E}{1-\nu^{2}}}$$

**Figure 2:**
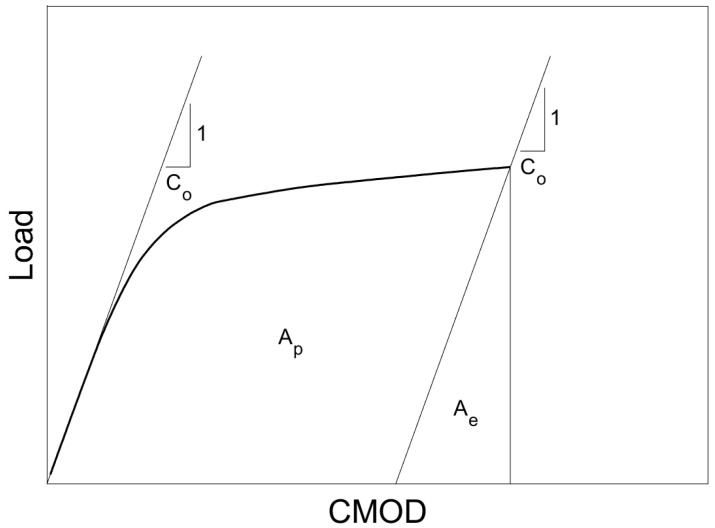
Schematic representation of load-CMOD curve for fracture initiation toughness.

A schematic of the load-CMOD curve identifying both plastic and elastic portions is presented in Figure 2. Both the load cell and clip gage used for fracture toughness testing met the resolution and data recording frequency requirements of ASTM E1921 [27]. Additionally, verification of clip gage accuracy was performed prior to each individual test.

### 2.3. Master Curve Analysis

Fracture toughness data obtained from the Charpy-sized SE(B) specimens were analyzed using the master curve methodology. Because the master curve considers the distribution of cleavage initiation sites throughout material thickness, fracture toughness is size-dependent. Thus, all fracture toughness data must be size corrected prior to reference temperature determination. Fracture toughness specimen thickness is defined using the *x*T nomenclature, where *x* is the specimen thickness in inches. It is typical to normalize fracture toughness results to a 1T specimen thickness. The adjustment in fracture toughness is given by Equation (3):

(3) KJcx=Kmin+KJco−KminBoBx14

where *B* is the specimen thickness, and *K_min_* is equal to 20 MPa√m (18 ksi√in). The subscript *x* is the desired specimen thickness to normalize to, and the subscript *o* is the specimen thickness tested. To convert to 1T fracture toughness, *B_x_* is equal to 25.4 mm (1.0 in).

The 1T fracture toughness data and respective test temperatures can then be used to calculate the provisional 1T master curve reference temperature, *T_oQ_*, using Equation (4):

(4) $$\overset{N}{\underset{i=1}{\sum }}\delta_{i}\frac{\exp\left[0.019\left(T_{i}-T_{oQ}\right)\right]}{11+77\exp\left[0.019\left(T_{i}-T_{oQ}\right)\right]}-\overset{N}{\underset{i=1}{\sum }}\frac{{\left(K_{Jc\left(i\right)}-20\right)}^{4}\exp\left[0.019\left(T_{i}-T_{oQ}\right)\right]}{{\left\{11+77\exp\left[0.019\left(T_{i}-T_{oQ}\right)\right]\right\}}^{5}}=0$$

where *N* is the number of specimens tested, *T_i_* is the test temperature in degrees Celsius corresponding to the *i*th specimen, *K_Jc(i)_* is the 1T fracture toughness in MPa√m corresponding to the *i*th specimen, and *δ_i_* is 1 if the *i*th specimen is uncensored and 0 if it is censored. Data can be censored by ductile crack growth that exceeds 0.05*b_o_*, in this case 0.25 mm (0.01 in), or by values of fracture toughness that are greater than the limit, *K_Jclimit_*. This size-dependent limit is intended to guarantee adequate levels of crack-tip constraint and is provided in Equation (5):

(5) $$K_{Jclimit}=\sqrt{\frac{Eb_{o}\sigma_{ys}}{30\left(1-\nu^{2}\right)}}$$

where *E* is the modulus of elasticity in MPa, *b_o_* is the initial remaining ligament in millimeters, *σ_ys_* is the material 0.2 percent offset yield stress in MPa, and *ν* is Poisson’s ratio for the material in question. Here, as well as throughout this article, the yield stress is taken to be the nominal yield stress of each grade, 485 and 690 MPa (70 and 100 ksi), respectively. Censored *K_Jc_* values still contribute to the calculation of the reference temperature but not to the validity of the dataset for dataset size requirements. *K_Jc_* values that exceed *K_Jclimit_* are instead replaced with the limit value for use in Equation (4), while the largest uncensored *K_Jc_* value from the dataset is substituted for crack-growth censored *K_Jc_* values.

Between six and eight valid and uncensored specimens tested at temperatures within ±50 °C (±90 °F) of the final reference temperature are required to constitute a valid dataset. Each specimen is assigned a weighting factor based on the difference between test temperature and reference temperature, or *T–T_o_*. If the sum of the weighted, valid, uncensored specimens is equal to or greater than 1.0, then the dataset is considered to be valid. The weighting factors for each of the three temperature ranges as prescribed by ASTM E1921 [10] are shown in Table 2. Weighting factors for specimens that are tested colder than the reference temperature are lower due to the assumption that they make a reduced accuracy contribution to calculation of the reference temperature.

The version of the fracture toughness standard used at the time of testing, ASTM E1921 [27], did not contain the lower-tail homogeneity screening procedure that has since been added to more recent versions of the standard. To align with current practices, the screening process is applied herein. The screening process is meant to determine whether the dataset is representative of a homogeneous material or if multiple behavior regimes are present. First, data above the median master curve are censored to the median, and a screened reference temperature, *T_o_*_,*scrn*_, is calculated. This is repeated until *T_o_*_,*scrn*_ for a given iteration is less than 0.5 °C (0.9 °F) warmer than the previous *T_o_*_,*scrn*_. The dataset is then considered homogeneous if it satisfies the inequality shown in Equation (6):

(6) $$T_{o,scrn}-T_{oQ}\leq 1.44\sqrt{\frac{\beta^{2}}{r}}$$

where *β* is a sample size uncertainty factor, and *r* is the number of uncensored data used to determine *T_oQ_*. To determine *β*, an equivalent value of the median toughness, *K_Jc(med)_^eq^*, is calculated using Equation (7). *β* has a value of 18.0 °C (32.4 °F) if *K_Jc(med)_^eq^* is greater than 83 MPa√m (75 ksi√in), 18.8 °C (33.8 °F) if *K_Jc(med)_^eq^* is between 83 and 66 MPa√m (75 and 60 ksi√in), and 20.1 °C (36.2 °F) otherwise.

(7) $$K_{Jc\left(med\right)}^{eq}=\frac{1}{r}\;\overset{r}{\underset{i=1}{\sum }}30+70\exp\left(0.019\left(T_{i}-T_{o}\right)\right)$$

If the inequality in Equation (6) is not satisfied, the data are considered to be inhomogeneous. For datasets with nine or fewer specimens, a conservative estimate based on the warmest single-data reference temperature, *T_oIN_*, is used in place of the provisional reference temperature, *T_oQ_*. This is useful for structural analysis and fitness-for-service applications but is overly conservative for the purposes of material characterization and was thus not used in the analysis contained herein. Instead, all inhomogeneous datasets, regardless of size, are reported using the screened reference temperature, *T_o_*_,*scrn*_, in place of *T_oQ_*. Bimodal and multimodal analysis is possible for datasets with 20 or more specimens but is not discussed in detail as it is not applicable to the data analyzed.

Once the reference temperature has been calculated, the median master curve, *K_Jc(med)_*, is constructed at a range of temperatures using Equation (8):

(8) $$K_{Jc\left(med\right)}=30+70\exp\left[0.019\left(T-T_{o}\right)\right]$$

where *T* and *T_o_* are in degrees Celsius, and *K_Jc(med)_* is in MPa√m. This curve represents a cumulative probability of failure of 50% based on the probabilistic distribution of flaws throughout a material thickness. Tolerance bounds corresponding to 5% and 95% cumulative probability of failure can also be constructed through the use of Equation (9):

(9) $$K_{Jc\left(0.xx\right)}=20+{\left[\ln\left(\frac{1}{1-0.xx}\right)\right]}^{1/4}\left\{11+77\exp\left[0.019\left(T-T_{o}\right)\right]\right\}$$

where 0.*xx* represents the selected cumulative probability of failure. For example, for the 5% tolerance bound, 0.*xx* is equal to 0.05.

### 2.4. Crack Arrest Testing

**Figure 3:**
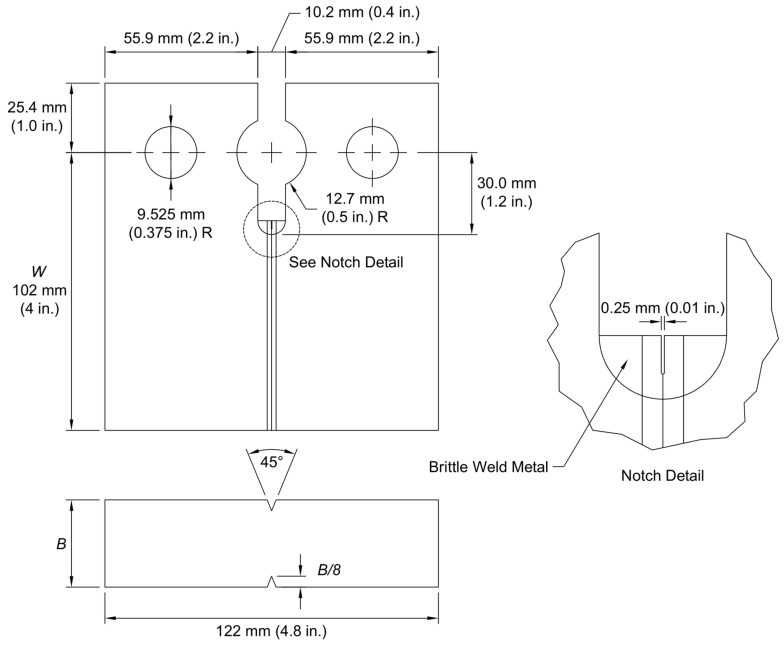
Crack arrest specimen geometry and notch detail.

Crack arrest specimens were fabricated and tested in accordance with ASTM E1221 [29]. The geometry of the specimens used, modified from typical C(T) specimens used in fracture toughness testing, is shown in Figure 3. Crack arrest specimens are typically full-thickness: thus, specimen thickness, *B*, is equal to the thickness of the plate sampled. The distance from the load line to the end of the specimen, *W*, is held constant at 102 mm (4 in) for all specimens. A wedge loading is applied at the center hole, which has a diameter of 25.4 mm (1.0 in) and is centered on a 10.2 mm (0.4 in) wide slot that extends 30 mm (1.2 in) past the load line into the specimen. The two 19 mm (0.75 in) diameter holes on either side of the slot are machined to accommodate clevis pins. A brittle weld is then deposited at the bottom of the slot using a hardfacing, or buildup, welding electrode. As with the SE(B) notches, an EDM is used to machine a 0.25 mm (0.01 in) wide notch in this weld. The initial crack length, *a_o_*, is nominally 28 mm (1.1 in) past the load line, approximately 2.5 mm (0.1 in) from base metal. Side grooves, centered on the slot with an included angle of 45 degrees, are employed and are similar to those used in SE(B) specimens. Each groove removes the outer eighth of the specimen; therefore, specimen net thickness, *B_N_*, is equal to 0.75*B*. Weld deposit, notch, and side-groove details are also shown in Figure 3.

**Figure 4:**
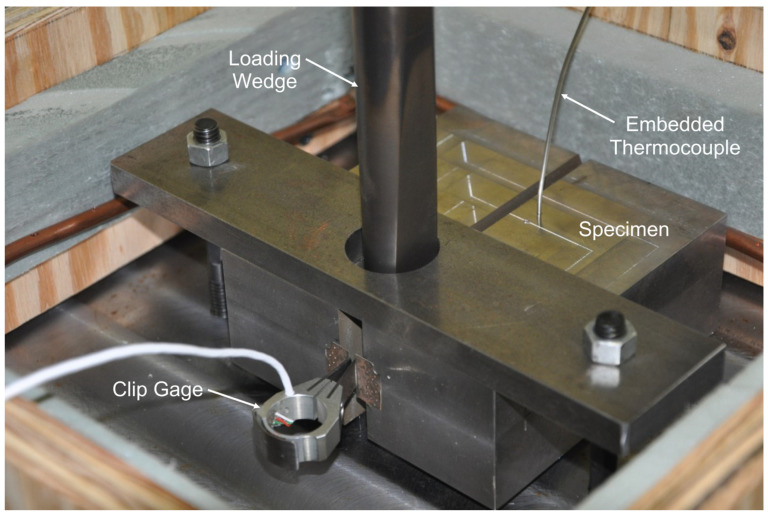
Crack arrest specimen seated in the test fixture.

Crack arrest testing was performed using an MTS Insight 150 kN (33 kip) electro-mechanical machine. The test chamber and liquid nitrogen temperature control system described previously was used to test crack arrest specimens at a range of sub-ambient temperatures. Wedge load was applied cyclically using a wedge and split pin apparatus as shown in Figure 4. Similar to the fracture initiation toughness testing described above, all measurement devices conformed to the resolution and data recording frequency requirements of ASTM E1221 [29].

CMOD was measured using a clip gage similar to the previously described SE(B) fracture toughness testing. Load was applied under displacement control at a rate of approximately 8 mm/min (0.3 in/min) until reaching the maximum, *δ_n_*_,*max*_, calculated using Equation (10):

(10) $$\delta_{n,max_{}}=\left[1+0.25\left(n-1\right)\right]\left[\frac{0.69\sigma_{ys}W\sqrt{B_{N}/B}}{Ef\left(a_{o}/W\right)}\right]$$

where *f(a_o_/W)* is a specimen geometry function that is defined in Equation (11), *n* is the cycle number, and all other variables are as previously defined.

(11) fao/W=1−aoW0.50.748−2.176aoW+3.56aoW2−2.55aoW3+0.62aoW4

**Figure 5:**
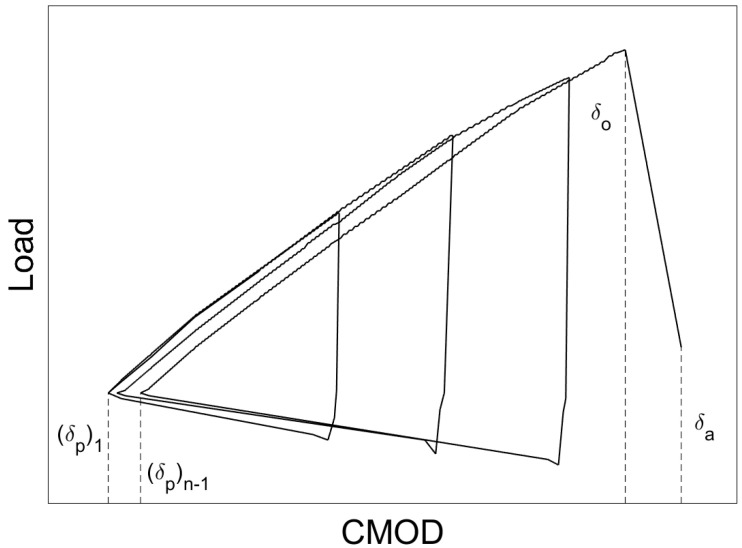
Schematic representation of load-CMOD curve for crack arrest toughness.

When the displacement limit for a given cycle was achieved, the wedge was removed from the specimen while CMOD was continuously recorded. The wedge was then reintroduced, and the next cycle of loading began. This process was repeated until either a fracture occurred or the maximum CMOD limit, *δ_o_*_,*limit*_, was reached. A schematic representation of the crack arrest cyclic loading protocol is presented in Figure 5.

The specified CMOD limit represents an amount of ductile tearing beyond which the specimen is unlikely to yield a valid result and is given in Equation (12):

(12) $$\delta_{o_{,limit}}=\left[\frac{1.5\sigma_{ys}W\sqrt{B_{N}/B}}{Ef\left(a_{o}/W\right)}\right]$$

When instability occurs and a propagating crack is arrested, crack arrest toughness, *K_Ia_*, is calculated based on specimen geometry; incremental CMOD values recorded during testing, *(δ_p_)*_1_ and *(δ_p_)_n−_*_1_; the CMOD value at crack initiation, *δ_o_*; and the CMOD value at crack arrest, *δ_a_*. However, if the CMOD limit was reached prior to fracture initiation, the specimen was re-machined to remove material in the plastic zone that formed around the initial notch. The test was then carried out again at a lower temperature in an attempt to initiate fracture. Crack arrest testing proved to be much more difficult than impact and fracture toughness testing. Of the 55 crack arrest specimens fabricated, only 41 fractured, and only 10 of these produced valid *K_Ia_* values.

### 2.5. Impact Energy–Fracture Toughness Correlations

As mentioned previously, a multitude of correlations to describe the relationship between impact energy and fracture toughness have been developed. In this research, three are evaluated: the Barsom and Rolfe Two-Stage Correlation [1] and the reference temperature estimates outlined by the British Standards (BS) 7910 [5] and the American Petroleum Institute (API) 579 [6]. A more comprehensive overview of relationships between fracture toughness and CVN impact energy can be found in Collins et al. [30].

Current material toughness standards for the United States steel bridge industry prescribed by the American Association of State Highway Transportation Officials (AASHTO) are based on the Barsom and Rolfe approach. The first step, a correlation between individual CVN energy values and dynamic fracture toughness, *K_dyn_*, is given in Equation (13):

(13) $$K_{dyn}=0.804\sqrt{E\cdot CVN}$$

where *K_dyn_* is the dynamic fracture toughness in MPa√m, *E* is the modulus of elasticity in GPa, and *CVN* is the impact energy in Joules. The effects of loading rate are then addressed by shifting *K_dyn_* values colder to an equivalent static fracture toughness, *K_st_*. This is accomplished using Equation (14):

(14) $$T_{shift}=119-0.12\sigma_{ys}$$

where *T_shift_* is the temperature shift from dynamic to static toughness in degrees Celsius. This correlation is limited to CVN values in the lower half of the temperature transition curve. Once shifted, the master curve method can be applied to the resulting *K_st_* values in the same manner as *K_Jc_* values, and a reference temperature estimate, *T_o_*_,*est*_, can be obtained.

By comparison, the BS and API methods are direct predictions of static reference temperature, *T_o_*_,*est*_. BS 7910 Method J.2.2 [5] utilizes the test temperatures that correspond to 27 and 40 J (20 and 30 ft-lbf), *T*_27__*J*_ and *T*_40__*J*_, respectively, to estimate reference temperature through the application of Equations (15) and (16):

(15) $$T_{o,est}=T_{27J}-18+T_{K}$$

(16) $$T_{o,est}=T_{40J}-24+T_{K}$$

where *T_K_* is a constant typically taken to be 25 °C (45 °F) that accounts for scatter in CVN results. If estimates from both equations are available, the reference temperature estimate is the average of the two. Similarly, API 579 “Fitness-For-Service” Annex 9F.4.3.2 [6] estimates reference temperature based on the test temperature corresponding to 28 J (20 ft-lbf), *T*_28__*J*_. If insufficient data are available to form a full temperature–transition curve, *T*_28__*J*_ is approximated using the following procedure.

First, the average full-size impact energy, *C_V_*, at the test temperature, *T*, is calculated. If impact energy data are available at multiple temperatures, the temperature at which *C_V_* is nearest to 28 J (20 ft-lbf) is chosen. Next, if direct measurements of lower and upper shelf impact energies, *C_V-LS_* and *C_V-US_*, are not available, they are estimated using Equations (17) and (18):

(17) $$C_{V-LS}=2.50+{\left(\frac{\sigma_{ys}}{460}\right)}^{2}$$

(18) $$C_{V-US}=\frac{C_{V}}{0.05+0.95{\left(\frac{SA}{100}\right)}^{0.72}}$$

where *C_V-LS_* and *C_V-US_* are lower and upper shelf impact energy in Joules, and *SA* is the percent shear area of the CVN fracture surface. If the shear area is not available, *C_V-US_* can be estimated as 200 J (147.6 ft-lbf) for steels with 0.01% sulfur or less, or 100 J (73.8 ft-lbf) for steels with a sulfur content greater than 0.01%.

If the hyperbolic tangent fitting parameter, *C*, is not known, it may be estimated based on the material yield stress and the measured or estimated upper shelf energy, *C_V-US_*. The value of *C* in degrees Celsius is estimated using Equation (19):

(19) $$C=34\;{}^{\circ}C+\frac{\sigma_{ys}}{35.1}-\frac{C_{V-US}}{14.3}$$

where all variables are as previously defined. The test temperature corresponding to 28 J (20 ft-lbf), *T*_28__*J*_, is then determined using Equation (20):

(20) $$T_{28J}=T-\beta C\cdot \ln\left[\frac{\left(C_{V}-C_{V-LS}\right)\cdot \left(C_{V-US}-28\right)}{\left(28-C_{V-LS}\right)\cdot \left(C_{V-US}-C_{V}\right)}\right]$$

Here, *β* equals 0.25 if *T* is greater than or equal to *T*_28__*J*_, 1.0 if *T* is less than *T*_28__*J*_, and 0.5 if the hyperbolic tangent fitting parameter *C* is known rather than estimated through Equation (19). The *β* factor ensures that this extrapolation is conservative due to the significant degree of uncertainty in the *C* parameter obtained from Equation (19). Finally, the master curve reference temperature estimate, *T_o_*_,*est*_, is obtained through the use of the estimated or known *T*_28__*J*_ and *C_V-US_* in Equation (21):

(21) $$T_{o,est}=T_{28J}-79\;{}^{\circ}C+\frac{\sigma_{ys}}{9}-\frac{C_{V-US}}{59}+\Delta T_{o}$$

where Δ*T_o_* is a temperature shift of +18 °C (+32.4 °F), which corresponds to one standard deviation on the reference temperature estimate from CVN data.

### 2.6. Stress Intensity Rate Adjustment

Both the BS and API reference temperature estimates are for a quasi-static rate fracture toughness. If estimates for reference temperature at intermediate or dynamic rates are desired, Equation (22) can be used to estimate an equivalent rate-adjusted reference temperature, *T_o_*_,*adj*_, from a known or estimated static reference temperature, *T_o_*_,*sta*_:

(22) $$T_{o,adj}=\frac{\left(T_{o,sta}+273.15\right)\Gamma }{\Gamma -\ln\dot{K}}-273.15$$

Here, *T_o_*_,*adj*_ and *T_o_*_,*sta*_ are both in degrees Celsius, $\dot{K}$ is the stress intensity rate in MPa√m/s, and *Γ* is defined by Equation (23), where all variables are as previously defined:

(23) $$\Gamma =9.9e\left[{\left(\frac{T_{o,sta}+273.15}{190}\right)}^{1.66}+{\left(\frac{\sigma_{ys}}{722}\right)}^{1.09}\right]$$

## 3. Results and Discussion

Although the current study focuses on the effects loading rate on fracture behavior, evaluating dynamic fracture initiation and crack arrest toughness, CVN impact energy and quasi-static fracture toughness results have been re-analyzed and are presented for context and comparison. Additional discussion and analysis of both CVN impact energy and quasi-static fracture toughness testing can be found in Collins et al. [17].

### 3.1. Charpy V-Notch Results

**Figure 6:**
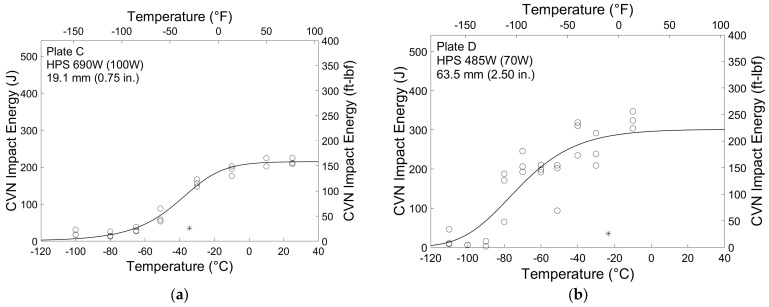
CVN impact test results for (**a**) Plate C and (**b**) Plate D.

Typical CVN test results from Plates C and D are shown in Figure 6. These results are representative of the behavior of the HPS 690 W and 485 W (100 W and 70 W) plates, respectively. The only exception is that Plates A and J did not display well-defined upper and lower energy shelves at the temperatures tested. Testing at more extreme temperatures could have resulted in more typical behavior exhibited by the other six plates. However, the behavior of the steel at AASHTO minimum service temperatures was desired, so more extreme test temperatures were not used. The solid line in the plots is the five-parameter sigmoid curve given in Equation (1) graphically representing the different behavior regimes of the material. The curve was fit to the data using the least squares method. Also shown is the fracture critical minimum average energy requirement for each grade as per the AASHTO LRFD BDS [7], represented by an asterisk. For both grades tested, the minimum average energy is 48 J (35 ft-lbf). This requirement is applied at −34 °C (−30 °F) for HPS 690 W (100 W) and at −23 °C (−10 °F) for HPS 485 W (70 W).

All plates clearly exhibit impact energy absorption that greatly exceeds the impact energy required in the AASHTO BDS [7] and ASTM A709 [31]. In fact, minimum average impact energy is also exceeded at the Zone III service temperature of −51 °C (−60 °F). Average impact energy at this temperature ranged from 66.7 to 98.1 J (49.2 to 72.3 ft-lbf) for HPS 690 W (100 W) plates and from 162 to 288 J (119 to 213 ft-lbf) for HPS 485 W (70 W) plates. The HPS 485 W (70 W) plates also display higher upper shelf energies and colder ductile-to-brittle transition temperatures than the HPS 690 W (100 W) plates.

Table 3 summarizes the temperatures at which the sigmoid curve is equal to 27 and 40 J (20 and 30 ft-lbf), *T*_27__*J*_ and *T*_40__*J*_, and the static reference temperature estimates, *T_o_*_,*est*_, from each of the three methods described above for all eight plates. It can be seen that for the HPS 690 W (100 W) plates, all three methods are in close agreement, with only a maximum of approximately 4 °C (7.2 °F) separating the three reference temperature estimates for a given plate. The range of estimates for the HPS 485 W (70 W) is much larger, with a maximum difference of approximately 39 °C (70.2 °F) between the Barsom and Rolfe and BS 7910 estimates for Plate I. This is likely caused by the increased scatter present in the HPS 485 W (70 W) results.

### 3.2. Fracture Toughness Results

**Figure 7:**
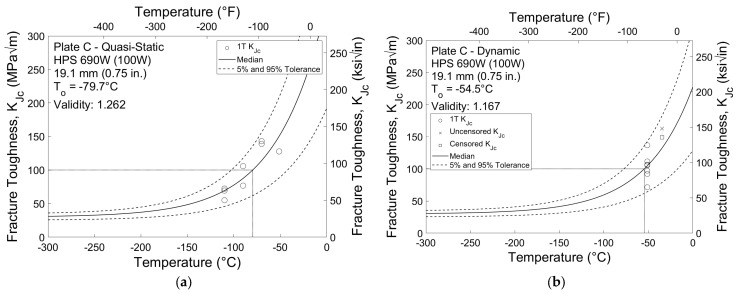
1T fracture toughness results for Plate C at (**a**) quasi-static and (**b**) dynamic stress intensity rates.

**Figure 8:**
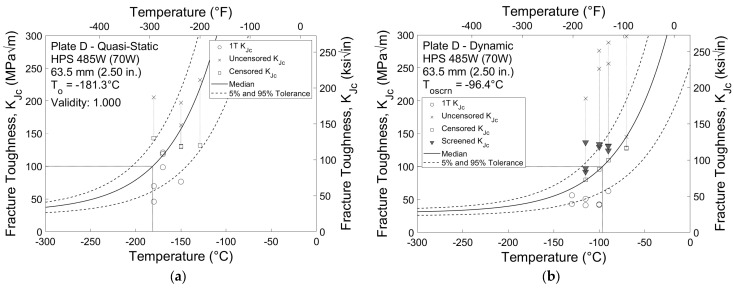
1T fracture toughness results for Plate D at (**a**) quasi-static and (**b**) dynamic stress intensity rates.

Fracture toughness test results that have been normalized to a 1T specimen thickness for Plates C and D are shown in Figure 7 and Figure 8, respectively. These results are typical for the HPS 690 W and 485 W (100 W and 70 W) plates, although the fracture toughness test results exhibit a slightly higher degree of variation than the impact test results. *K_Jc_* values that are not crack growth or limit censored are represented by circles, while values that are crack growth or limit censored are represented by an X. For datasets that fail the homogeneity screening criterion shown in Equation (6), such as in Figure 8b, screening censored values are shown by a downwards triangle. In all cases, censored *K_Jc_* values are represented by squares.

In total, four of the sixteen datasets were considered inhomogeneous: specimens tested at the dynamic rate sampled from Plates D and H and both specimens sampled from Plate I tested at both quasi-static and dynamic rates. Table 4 summarizes the reference temperatures calculated for each of the sixteen fracture toughness datasets. For the twelve homogeneous datasets, validity and *T_o_* are reported, while the screened reference temperature *T_o_*_,*scrn*_ is reported for the four inhomogeneous datasets.

Similar to the results from the CVN impact tests, specimens sampled from the HPS 485 W (70 W) plates exhibit greater fracture toughness than those sampled from the HPS 690 W (100 W) plates. The average reference temperature for HPS 485 W (70 W) is approximately −162 °C (−260 °F) for quasi-static tests and −87 °C (−124 °F) for dynamic tests. This is significantly colder than the average reference temperatures for HPS 690 W (100 W) of −92 °C (−134 °F) for quasi-static tests and −55 °C (−68 °F) for dynamic tests. There is also a greater difference between reference temperatures obtained from the two stress intensity rates for the HPS 485 W (70 W) specimens. The average experimental temperature shift between quasi-static and dynamic reference temperatures for HPS 485 W (70 W) is 75 °C (135 °F), compared to 37 °C (67 °F) for HPS 690 W (100 W).

### 3.3. Crack Arrest Results

**Figure 9:**
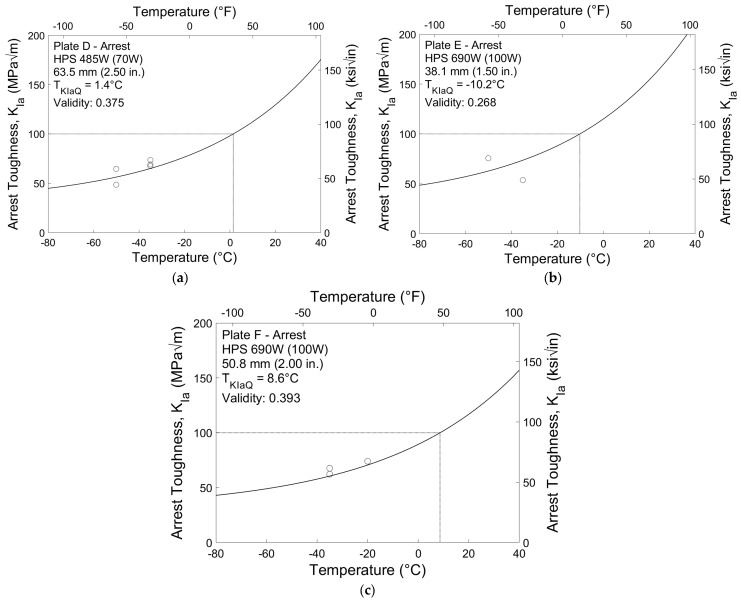
Crack arrest test results for (**a**) Plate D, (**b**) Plate E, and (**c**) Plate F.

Valid crack arrest toughness results analyzed using the master curve methodology are shown in Figure 9. Unlike the tolerance bounds for fracture initiation toughness shown in Figure 7 and Figure 8, crack arrest toughness tolerance bounds are based on the scatter of the data rather than probabilistic flaw distribution. While there is little scatter in the valid results presented, there are insufficient data to constitute valid datasets and create tolerance bounds. As such, only the median curve is shown. Due to the relatively low degree of scatter in the results shown, the provisional arrest reference temperatures calculated for the three plates, *T_KIaQ_*, appear to be reasonable estimates despite having validity factors below 0.5. The reference temperatures range from −10 to 8.6 °C (14 to 47 °F), significantly warmer than the fracture toughness reference temperatures presented above.

**Figure 10:**
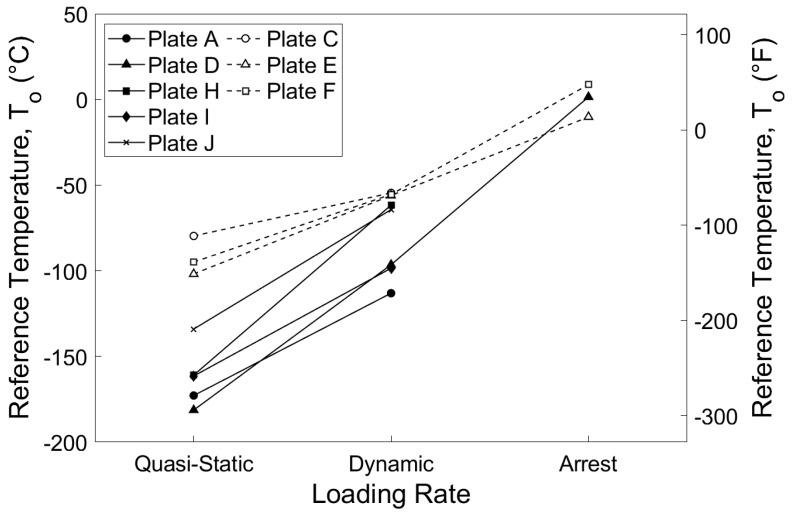
Reference temperature comparison.

A comparison between *T_o_*_,*sta*_, *T_o_*_,*dyn*_, and *T_KIaQ_* is provided in Figure 10. Results from the five HPS 485 W (70 W) plates are shown with solid lines and symbols, and the three HPS 690 W (100 W) plates have dashed lines and open symbols.

The results indicate that the rate effect is slightly more pronounced for HPS 485 W (70 W) than HPS 690 W (100 W), evidenced by the steeper slope from quasi-static to dynamic reference temperature. While there are limited crack arrest data available, it appears that the difference between *T_o_*_,*dyn*_ and *T_KIaQ_* is similar in magnitude to the difference between *T_o_*_,*sta*_ and *T_o_*_,*dyn*_. The grade HPS 485 W (70 W) Plate D exhibits a greater change from fracture to arrest toughness than the grade HPS 690 W (100 W) Plates E and F. From these limited results, a preliminary correlation between *T_o_*_,*sta*_ and *T_KIaQ_* is presented in Equation (24):

(24) $$\Delta T_{arrest}=\frac{27600}{\sigma_{ys}-207}$$

where Δ*T_arrest_* is an experimental temperature shift in degrees Celsius, and *σ_ys_* is the material 0.2 percent offset yield stress in MPa. This correlation incorporates the observed inverse relationship between yield stress and the difference between fracture and arrest toughness. The values of 207 and 27,600 are constants chosen to provide the best fit to the data available. Resulting values of Δ*T_arrest_* are 99 °C (179 °F) for HPS 485 W (70 W) and 57 °C (103 °F) for HPS 690 W (100 W). This closely matches the experimentally obtained differences between *T_o_*_,*sta*_ and *T_KIaQ_* of 98 °C (176 °F) for Plate D, 46 °C (83 °F) for Plate E, and 64 °C (115 °F) for Plate F.

### 3.4. Reference Temperature Rate Adjustment

For purposes of calculating an estimated dynamic reference temperature, *T_o_*_,*adj*_, from a known static reference temperature, *T_o_*_,*sta*_, using Equation (22), the stress intensity rate $\dot{K}$ was taken as the mean of the individual stress intensity rates for each of the eight plates. Mean stress intensity rates ranged from 2720 to 3650 MPa√m/s (2470 to 3320 ksi√in/s) and are summarized in Table 5. The difference between *T_o_*_,*sta*_ and *T_o_*_,*adj*_ can be considered to be a theoretical rate-dependent temperature shift, hereafter referred to as Δ*T_theoretical_*. This temperature shift, as well as the temperature shift prescribed by the second stage of the Barsom and Rolfe method shown in Equation (14), *T_shift_*, and the experimental temperature shift between quasi-static and dynamic refence temperatures contained in Table 4, Δ*T_experimental_*, are shown in Table 5. It should be noted that the large Δ*T_experimental_* for Plates D and H is in part due to the dynamic reference temperatures for these plates being screened to the warmer *T_o_*_,*scrn*_ while the quasi-static reference temperatures are not. Because both quasi-static and dynamic reference temperatures for Plate I are screened, the difference is more similar to Plates A and J.

Of the two temperature shifts evaluated, the Barsom and Rolfe *T_shift_* is consistently greater in magnitude than Δ*T_theoretical_* by approximately 10 °C (18 °F) for HPS 690 W (100 W) and by 18 °C (32 °F) for HPS 485 W (70 W). The Barsom and Rolfe temperature shift is reasonably accurate for all plates except for Plates D and H, likely due to the use of screened reference temperatures mentioned above. The average difference between *T_shift_* and Δ*T_experimental_,* not including Plates D and H, is 1.9 °C (3.4 °F), with a maximum error of 11 °C (20 °F). The theoretical temperature shift from Equation (22) closely matches the experimental temperature shift for Plate C but underestimates the difference between quasi-static and dynamic reference temperature for all other plates. In this case, an underestimation of temperature shift results in an estimated dynamic reference temperature, *T_o_*_,*adj*_, that is colder than the experimentally determined dynamic reference temperature, *T_o_*_,*dyn*_. Because colder reference temperatures correspond to increased material fracture toughness, this error results in an unconservative estimate of dynamic fracture toughness.

### 3.5. Correlation Evaluations

The experimental reference temperatures shown in Table 4 and the reference temperature estimates from the three correlation methods presented in Table 3 are summarized for comparison in Table 6. The dynamic reference temperature estimates using the Barsom and Rolfe correlation are obtained by calculating a reference temperature using *K_dyn_* estimates obtained using Equation (13) and neglecting the temperature shift of Equation (14). Dynamic reference temperature estimates for the BS 7910 and API 579 correlations adjust static estimates based on experimental stress intensity rate using the rate adjustment shown in Equation (22).

**Figure 11:**
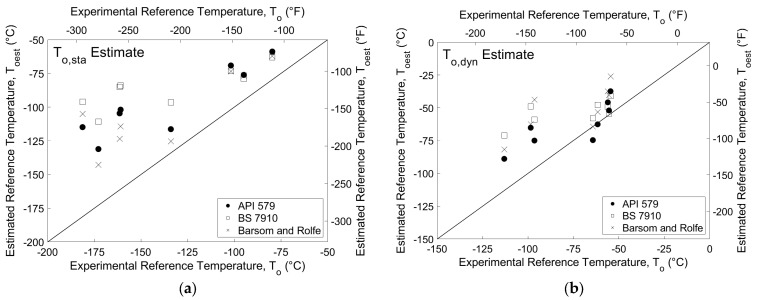
Experimental and estimated (**a**) static and (**b**) dynamic reference temperatures.

The majority of reference temperature estimates are warmer than the experimental reference temperatures, indicating that all three correlations are generally conservative for the specimen sizes and steel grades tested. The Barsom and Rolfe quasi-static estimates are warmer by an average of 33 °C (59 °F) for all plates. Comparatively, the BS 7910 and API 579 quasi-static estimates are warmer than the experimental values by an average of 50 °C (90 °F) and 39 °C (71 °F), respectively. Dynamic estimates of reference temperature are closer to the experimental values than static estimates, with average errors of 24, 21, and 12 °C (43, 38, and 22 °F) for the Barsom and Rolfe, BS 7910, and API 579 correlations, respectively. The difference between experimental and estimated reference temperature is also expressed visually in Figure 11. The diagonal line represents an exact match between the two, with points above the line corresponding to a conservative estimate of reference temperature. It should be noted that the axes for the two plots presented in Figure 11 have different scales for the purposes of data presentation. However, the range of values for each plot is the same, so performance relative to the ideal correlation line can be directly compared. Results indicate that each of the three correlations analyzed provide conservative estimates of fracture toughness for Charpy-sized SE(B) specimens tested at both quasi-static and dynamic rates, with the exception of the API 579 dynamic reference temperature estimates for Plates H and J and the Barsom and Rolfe dynamic reference temperature estimate for Plate J.

## 4. Conclusions

Results of the CVN impact tests indicate that all plates greatly exceed the required minimum average impact energy prescribed by AASHTO. CVN impact energy results exceed these levels not only at the required fracture critical test temperatures of −23 and −34 °C (−10 and −30 °F), but also at the Zone III service temperature of −51 °C (−60 °F). Average impact energy at −51 °C (−60 °F) ranged from 67 to 98 J (49 to 72 ft-lbf) for HPS 690 W (100 W) plates and from 162 to 288 J (119 to 213 ft-lbf) for HPS 485 W (70 W) plates. In general, the HPS 485 W (70 W) impact specimens exhibited colder ductile-to-brittle transition temperatures and greater upper shelf energy absorption than the HPS 690 W (100 W) impact specimens.

Analysis of Charpy-sized fracture toughness tests resulted in average quasi-static master curve reference temperatures of approximately −162 °C (−260 °F) and −92 °C (−134 °F) for grades HPS 485 W and 690 W (70 W and 100 W), respectively. Average dynamic master curve reference temperatures for the two grades were −87 °C (−124 °F) for HPS 485 W (70 W) and −55 °C (−68 °F) for HPS 690 W (100 W). Similar to the impact test results, both grades tested exhibited high levels of fracture toughness, with all experimental reference temperatures being colder than the Zone III service temperature of −51 °C (−60 °F). Of the two grades tested, it appears that the rate effect is more pronounced in the HPS 485 W (70 W). The theoretical temperature shift between quasi-static and dynamic stress intensity rates predicted colder dynamic reference temperatures than were experimentally determined, indicating that the rate-dependent adjustment is unconservative for the grades and specimen sizes tested. The yield stress-dependent temperature shift developed by Barsom and Rolfe more accurately reflects the difference between the quasi-static and dynamic fracture toughness results, with an average error between Δ*T_experimental_* and *T_shift_* for all plates, excluding D and H, of 1.9 °C (3.4 °F).

Crack arrest testing had minimal success, with only 10 valid *K_Ia_* values of the 55 specimens fabricated and tested. Due to the small dataset sizes, valid arrest toughness reference temperatures were not obtained. The estimated crack arrest reference temperatures were between −10 and 9 °C (14 and 47 °F). A correlation between quasi-static and arrest reference temperature in the form of a temperature shift is presented. This correlation is based on the observed inverse relationship between yield stress and the difference between fracture initiation and arrest toughness. The proposed correlation may be used by engineers to estimate the crack arrest capability of a material when only fracture initiation information is available.

Correlations between impact energy and fracture toughness, which form the basis for current material screening practices in the steel bridge industry, are shown to be conservative for the grades of A709 HPS and the specimen sizes tested. The Barsom and Rolfe Two-Stage Correlation estimates static reference temperatures that are approximately 30 °C (54 °F) warmer than experimentally obtained values. The BS 7910 and API 579 methods are similarly conservative, with static reference temperature estimates warmer than experimental values by an average of 50 °C (90 °F) and 39 °C (71 °F), respectively. This supports the use of CVN impact testing to verify adequate toughness of A709 HPS base metals for fracture critical structural applications.

Although the research presented herein represents an advancement in terms of understanding and characterizing rate effects on the fracture behavior of HPS, challenges to the implementation of high-toughness materials as an approach to mitigate in-service inspection requirements remain. These challenges should be addressed in future studies and include characterizing the behavior and possible controlling influence of weld metal and heat-affected zone fracture toughness on the reliability of the fracture limit state.

## Figures and Tables

**Table 1:** Details of HPS plates and specimen counts.

Plate	Grade MPa (ksi)	Thickness mm (in)	CVN Impact	Charpy-Sized SE(B)		Crack Arrest
				Static	Dynamic	
A	485 (70)	25.4 (1.00)	24 (24)	9 (8)	11 (10)	-
C	690 (100)	19.1 (0.75)	24 (24)	8 (8)	8 (8)	-
D	485 (70)	63.5 (2.50)	24 (23)	13 (10)	17 (17)	10 (5)
E	690 (100)	38.1 (1.50)	24 (23)	15 (12)	13 (13)	5 (2)
F	690 (100)	50.8 (2.00)	24 (24)	15 (11)	12 (12)	3 (3)
H	485 (70)	31.8 (1.25)	24 (24)	10 (10)	8 (8)	-
I	485 (70)	31.8 (1.25)	24 (24)	9 (8)	8 (8)	-
J	485 (70)	38.1 (1.50)	24 (23)	8 (8)	10 (10)	-

**Table 2:** Validity weighting factors.

Temperature Range, *T*–*T_o_* °C (°F)	Weighting Factor
+50 to −14 (+90 to −25)	1/6
−15 to −35 (−27 to −63)	1/7
−36 to −50 (−65 to −90)	1/8

**Table 3:** Summary of CVN impact results.

Grade MPa (ksi)	Plate	*T_27J_* °C (°F)	*T_40J_* °C (°F)	*T_o_*_,*est*_, °C (°F)		
				B and R	BS 7910	API 579
690 (100)	C	−71 (−96)	−63 (−81)	−62 (−80)	−63 (−81)	−59 (−74)
	E	−81 (−113)	−73 (−100)	−73 (−100)	−73 (−99)	−69 (−93)
	F	−88 (−127)	−78 (−108)	−77 (−106)	−79 (−110)	−76 (−105)
485 (70)	A	−119 (−183)	−110 (−166)	−143 (−225)	−111 (−167)	−131 (−204)
	D	−103 (−153)	−98 (−144)	−105 (−157)	−96 (−141)	−115 (−175)
	H	−89 (−129)	−86 (−124)	−114 (−174)	−84 (−119)	−102 (−151)
	I	−91 (−132)	−87 (−124)	−124 (−191)	−85 (−121)	−105 (−156)
	J	−104 (−156)	−97 (−142)	−125 (−194)	−97 (−142)	−116 (−177)

**Table 4:** Summary of master curve reference temperatures.

Grade MPa (ksi)	Plate	Stress Intensity Rate	Validity	Reference Temperature °C (°F)
690 (100)	C	Quasi-Static	1.262	−80 (−111)
		Dynamic	1.167	−55 (−66)
	E	Quasi-Static	1.476	−102 (−151)
		Dynamic	1.833	−56 (−69)
	F	Quasi-Static	1.214	−95 (−139)
		Dynamic	1.619	−55 (−68)
485 (70)	A	Quasi-Static	1.000	−173 (−279)
		Dynamic	1.500	−113 (−172)
	D	Quasi-Static	1.000	−181 (−294)
		Dynamic	-	−96 (−142)
	H	Quasi-Static	1.000	−161 (−258)
		Dynamic	-	−62 (−79)
	I	Quasi-Static	-	−161 (−259)
		Dynamic	-	−98 (−145)
	J	Quasi-Static	1.095	−134 (−209)
		Dynamic	1.196	−64 (−84)

**Table 5:** Rate-dependent reference temperature shifts.

Grade MPa (ksi)	Plate	Mean Stress Intensity Rate, $\dot{K}$ MPa√m/s (ksi√in/s)	Δ*T_theoretical_* °C (°F)	*T_shift_* °C (°F)	Δ*T_experimental_* °C (°F)
690 (100)	C	2720 (2470)	24 (43)	36 (65)	25 (45)
	E	3270 (2980)	27 (49)	36 (65)	46 (82)
	F	3230 (2940)	26 (47)	36 (65)	40 (71)
485 (70)	A	3020 (2750)	43 (78)	61 (110)	60 (108)
	D	2820 (2570)	42 (75)	61 (110)	85 (153)
	H	3540 (3220)	45 (81)	61 (110)	99 (179)
	I	3350 (3050)	44 (80)	61 (110)	63 (113)
	J	3650 (3320)	44 (79)	61 (110)	70 (126)

**Table 6:** Experimental and estimated fracture toughness reference temperatures.

Grade MPa (ksi)	Plate	Stress Intensity Rate	*T_o_* °C (°F)	*T_o_*_,*est*_ °C (°F)		
				B and R	BS 7910	API 579
690 (100)	C	Quasi-Static	−80 (−111)	−62 (−80)	−63 (−81)	−59 (−74)
		Dynamic	−55 (−66)	−26 (−15)	−41 (−42)	−37 (−35)
	E	Quasi-Static	−102 (−151)	−73 (−100)	−73 (−99)	−69 (−93)
		Dynamic	−56 (−69)	−37 (−35)	−49 (−57)	−46 (−50)
	F	Quasi-Static	−95 (−139)	−77 (−106)	−79 (−110)	−76 (−105)
		Dynamic	−55 (−68)	−40 (−41)	−54 (−66)	−52 (−62)
485 (70)	A	Quasi-Static	−173 (−279)	−143 (−225)	−111 (−167)	−131 (−204)
		Dynamic	−113 (−172)	−82 (−115)	−71 (−96)	−89 (−128)
	D	Quasi-Static	−181 (−294)	−105 (−157)	−96 (−141)	−115 (−175)
		Dynamic	−96 (−142)	−44 (−47)	−59 (−74)	−75 (−103)
	H	Quasi-Static	−161 (−258)	−114 (−174)	−84 (−119)	−102 (−151)
		Dynamic	−62 (−79)	−53 (−64)	−48 (−54)	−63 (−81)
	I	Quasi-Static	−161 (−259)	−124 (−191)	−85 (−121)	−105 (−156)
		Dynamic	−98 (−145)	−63 (−81)	−49 (−56)	−65 (−85)
	J	Quasi-Static	−134 (−209)	−125 (−194)	−97 (−142)	−116 (−177)
		Dynamic	−64 (−84)	−64 (−84)	−58 (−72)	−75 (−102)

## Data Availability

The majority of data presented in this study are openly available in [14,15]. Additional information is available upon request.

## Notation

The following symbols are used in this paper:

*a*	Asymptotic minimum of sigmoid fit;
*a_o_*	Initial crack size;
*A*	Total area under load-CMOD curve;
*A_e_*	Elastic component of area under load-CMOD curve;
*A_p_*	Plastic component of area under load-CMOD curve;
*b*	Asymptotic maximum of sigmoid fit;
*b_o_*	Initial remaining ligament size;
*B*	Specimen thickness;
*B_N_*	Net specimen thickness;
*B_o_*	Initial specimen thickness used in thickness adjustment;
*B_x_*	Desired specimen thickness for thickness adjustment;
*c*	Location parameter of sigmoid fit;
*C(T)*	Compact specimen geometry;
*C_o_*	Compliance, the reciprocal of the initial elastic slope;
*C_V_*	Average CVN impact energy used in API 579 correlation approach;
*CVN(T)*	Charpy V-notch impact energy at a given temperature, T;
*C_V-LS_*	Charpy V-notch impact energy at lower shelf behavior;
*C_V-US_*	Charpy V-notch impact energy at upper shelf behavior;
*d*	Slope parameter of sigmoid fit;
*E*	Modulus of elasticity;
*J_c_*	Critical value of J-integral at failure;
*J_e_*	Elastic component of J-integral calculation;
*J_p_*	Plastic component of J-integral calculation;
$\dot{K}$	Stress intensity rate;
*K_c_*	Critical fracture toughness;
*K_dyn_*	Dynamic fracture toughness;
*K_e_*	Elastic fracture toughness;
*K_Ia_*	Crack arrest fracture toughness;
*K_Jc_*	Elastic–plastic equivalent fracture toughness converted from critical J-integral;
*K_Jc(0.xx)_*	Elastic–plastic fracture toughness tolerance limit of xx per cent;
*K_Jc(i)_*	Elastic–plastic fracture toughness of ith specimen;
*K_Jclimit_*	Elastic–plastic fracture toughness limit in master curve analysis;
*K_Jc(med)_*	Median elastic–plastic fracture toughness;
*K_Jc(med)_^eq^*	Equivalent median toughness used in inhomogeneity screening process
*K_Jc(o)_*	Initial elastic–plastic fracture toughness used in thickness adjustment;
*K_Jc(x)_*	Size adjusted fracture toughness for specimen of thickness B_N_;
*K_min_*	Absolute minimum fracture toughness equal to 20 MPa√m (18 ksi√in);
*m*	Scale parameter of sigmoid fit;
*n*	Load cycle count in crack arrest testing;
*r*	Number of uncensored data used to determine provisional reference temperature;
*SA*	Percent shear area of CVN fracture surface;
*SE(B)*	Single edge bend specimen geometry;
*T*	Test temperature;
*T*_27__*J*_	Test temperature corresponding to CVN of 27 J;
*T*_28__*J*_	Test temperature corresponding to CVN of 28 J;
*T*_40__*J*_	Test temperature corresponding to CVN of 40 J;
*T_i_*	Test temperature of ith specimen;
*T_k_*	Temperature adjustment based on CVN scatter in BS 7910;
*T_KIaQ_*	Provisional arrest reference temperature;
*T_o_*	Reference temperature where median toughness equals 100 MPa√m (91 ksi√in);
*T_o,adj_*	Rate adjusted reference temperature;
*T_o,est_*	Prediction of static reference temperature correlated from CVN data;
*T_oQ_*	Provisional reference temperature prior to validation;
*T_o,scrn_*	Reference temperature following inhomogeneity screening procedure;
*T_o,sta_*	Quasi-static rate reference temperature;
*T_shift_*	Temperature shift rate adjustment in Barsom and Rolfe Two-Stage correlation;
*W*	Specimen width;
*x*T	Specimen thickness designation with x in inches;
*β*	Sample size uncertainty factor used in inhomogeneity screening process;
*δ_a_*	CMOD corresponding to crack arrest;
*δ_i_*	Validity of ith specimen in master curve analysis, valid = 1, invalid = 0;
*δ_n,max_*	Maximum CMOD limit for a given load cycle in crack arrest testing;
*δ_o_*	CMOD corresponding to crack initiation in crack arrest testing;
*δ_o,limit_*	Overall CMOD limit for crack arrest testing;
*(δ_p_)_1_*	CMOD offset at the end of the first crack arrest load cycle;
*(δ_p_)_n−1_*	CMOD offset at the beginning of the last crack arrest load cycle;
Δ*T_experimental_*	Difference between quasi-static and dynamic reference temperature;
Δ*T_theoretical_*	Theoretical rate-dependent shift applied to reference temperature;
*Γ*	Material fitting coefficient used in Wallin rate adjustment;
*σ_ys_*	0.2% offset yield strength;
*ν*	Poisson’s ratio.
